# Identifying components of the hair-cell interactome involved in cochlear amplification

**DOI:** 10.1186/1471-2164-10-127

**Published:** 2009-03-25

**Authors:** Jing Zheng, Charles T Anderson, Katharine K Miller, MaryAnn Cheatham, Peter Dallos

**Affiliations:** 1 Department of Communication Sciences and Disorders, The Hugh Knowles Center, Northwestern University, Evanston, IL 60208, USA; 2 Department of Neurobiology and Physiology, Northwestern University, Evanston, IL 60208, USA

## Abstract

**Background:**

Although outer hair cells (OHCs) play a key role in cochlear amplification, it is not fully understood how they amplify sound signals by more than 100 fold. Two competing or possibly complementary mechanisms, stereocilia-based and somatic electromotility-based amplification, have been considered. Lacking knowledge about the exceptionally rich protein networks in the OHC plasma membrane, as well as related protein-protein interactions, limits our understanding of cochlear function. Therefore, we focused on finding protein partners for two important membrane proteins: Cadherin 23 (cdh23) and prestin. Cdh23 is one of the tip-link proteins involved in transducer function, a key component of mechanoelectrical transduction and stereocilia-based amplification. Prestin is a basolateral membrane protein responsible for OHC somatic electromotility.

**Results:**

Using the membrane-based yeast two-hybrid system to screen a newly built cDNA library made predominantly from OHCs, we identified two completely different groups of potential protein partners using prestin and cdh23 as bait. These include both membrane bound and cytoplasmic proteins with 12 being *de novo *gene products with unknown function(s). In addition, some of these genes are closely associated with deafness loci, implying a potentially important role in hearing. The most abundant prey for prestin (38%) is composed of a group of proteins involved in electron transport, which may play a role in OHC survival. The most abundant group of cdh23 prey (55%) contains calcium-binding domains. Since calcium performs an important role in hair cell mechanoelectrical transduction and amplification, understanding the interactions between cdh23 and calcium-binding proteins should increase our knowledge of hair cell function at the molecular level.

**Conclusion:**

The results of this study shed light on some protein networks in cochlear hair cells. Not only was a group of *de novo *genes closely associated with known deafness loci identified, but the data also indicate that the hair cell tip link interacts directly with calcium binding proteins. The OHC motor protein, prestin, also appears to be associated with electron transport proteins. These unanticipated results open potentially fruitful lines of investigation into the molecular basis of cochlear amplification.

## Background

**Figure 1:**
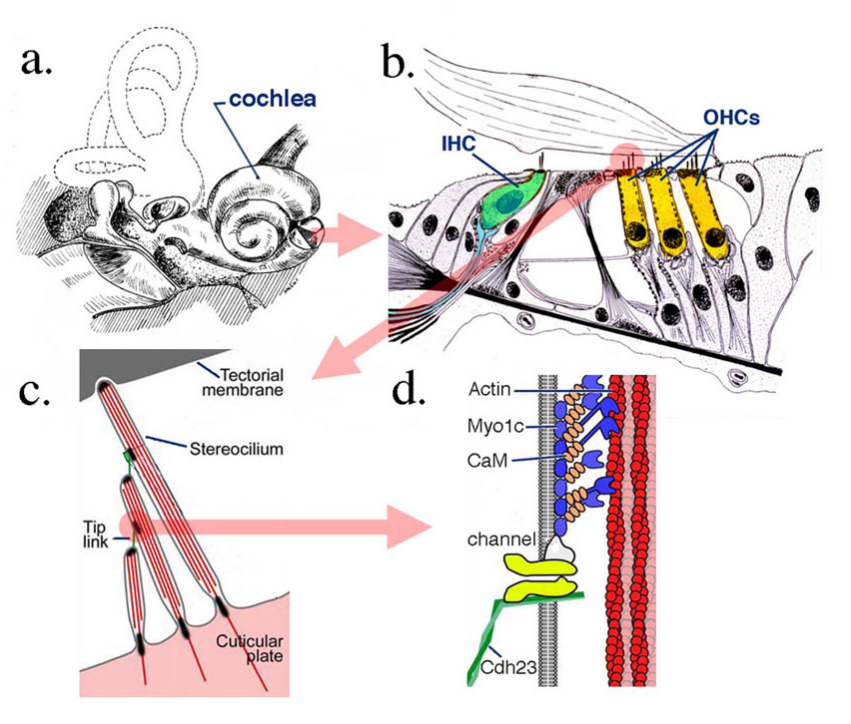
**Anatomical details of inner ear, cochlea and organ of Corti, the sense organ of mammalian hearing**. The cochlea, a fluid-filled tripartite channel, is located in the inner ear (**a**). A hemisected cochlea provides a radial view of the organ of Corti, a cellular matrix showing the location of hair cells. IHC: inner hair cell OHC: outer hair cell (**b**). The input organelles of hair cells, the stereocilia, are connected by different links, including tip-link proteins allowing movement as a unit. Deflection of the stereocilary bundle due to displacement between the top of the organ of Corti and the bottom of the tectorial membrane provides tension to the tip link, which, in turn, modulates the MET channel's open probability(**c**). The tip link is partially composed of cdh23, which is presumed to interact with the MET channel (**d**) either directly or indirectly. Images in (**c**) and (**d**) are modified from LeMasurier and Gillespie [33]. Myo1c: myosin 1c, CaM: calmodulin.

Hearing impairment is the most common sensory defect, affecting millions of people ranging from newborns to the elderly. Causes of hearing impairment are often associated with damage to one or both types of hair cells (Figure 1): inner hair cells (IHCs) and/or outer hair cells (OHCs). Both mechanoreceptor cell populations are housed in the mammalian organ of Corti (OC), a cellular matrix within the cochlea (Figure 1). Each hair cell has a staircase array of stereocilia (actin-filled villi) located at the apical surface of the cell body. Several different kinds of extracellular links connect individual stereocilia into a bundle, allowing the structure to move as a unit in response to mechanical stimulation [1-5]. A tip link connects the top of each shorter stereocilium to the side of its taller neighbor [6]. Vibrations of the basilar membrane result in deflection of the hair bundles, which modulate tension on the tip links, thereby controlling the open probability of cation-selective mechanoelectrical transduction (MET) channels [6,7]. Cations, principally K^+ ^and Ca^++^, flow through MET channels and ultimately change the membrane potential. In IHCs the membrane potential facilitates afferent neurotransmitter release. Hence, these cells are considered the true sensory receptors for hearing (for review, see [8]). In contrast, OHCs undergo rapid somatic length changes when the voltage across their basolateral membranes is altered [9-12]. This somatic electromotility is thought to function as part of the cochlear amplifier by providing local mechanical enhancement of the basilar membrane's vibratory pattern [8]. Without OHCs, hearing threshold is elevated by 40–50 dB [13], frequency resolution deteriorates [14] and the ear's operation is linearized [15].

Prestin is the molecule responsible for generating OHC somatic electromotility [16]. Prestin proteins are abundantly expressed in the OHC basolateral membrane [17] and are known to underlie somatic electromotility [16]. Prestin knockout (KO) mice show a loss of OHC electromotility, an increase in hearing threshold of ~50 dB, and a loss of frequency selectivity. In the prestin-KO model, OHCs are shorter than normal, and progressive OHC death is observed [18,19]. In order to eliminate potential deleterious effects due to the anatomical changes, two prestin knock-in (KI) mouse models have been created: C1 KI and 499 KI. C1-KI mice carry an altered but fully functional prestin: C1-mutant [20]. C1-KI mice have normal cochlear amplification and show no OHC loss [21]. In contrast, 499-KI mice carry a V409H/Y501H mutation in which prestin loses almost all motile function but retains its ability to target the plasma membrane (PM) [22]. Even so, progressive OHC death is still found in the 499-KI mice [23]. To restate: OHCs that lack prestin, as well as OHCs that lack fully *functional *prestin, show significant cell death due to some unknown mechanism. Although the functions of prestin-associated proteins may provide insights into OHC death, little is known regarding interactions between prestin and other proteins.

In order for the OHC's motor action to affect peripheral auditory function, a normal transduction of mechanical into electrical signals by the MET apparatus located at the tips of the stereocilia is required. Similar to other sensory systems [24], this MET apparatus is a complex composed of several proteins organized in an elegant and sophisticated fashion [25]. Mutations of these proteins cause damage to stereocilia and result in deafness (for review see [26]). By using various experimental methods and different species ranging from zebrafish to human, many components likely to be associated with the MET apparatus have been identified, including cdh23, myosin1c, protocadherin 15 (PCDH15) and harmonin (for review see [27]). However, additional important elements, including the MET channel protein itself, remain unknown. It is also known that the MET apparatus gives rise to active hair-bundle motility, indicating that it is capable of exerting forces to amplify mechanical stimuli [28-31]. This force was suggested to arise from myosin1c motors involved in slow adaptation and from the Ca^++^-dependent reclosure of MET channels (fast adaptation) (for review, see [27,32,33]. However, in spite of several proposed models [33], the mechanism for fast adaptation is not fully understood. In order to understand the association between fast adaptation and amplification, it is essential to know where Ca^++ ^action occurs. Several Ca^++^-dependent mechanisms for fast adaptation have been proposed (for review, see [27,33]). For example, Ca^++ ^could bind directly to the transduction channel [34,35]. Alternatively, Ca^++ ^could bind to an intracellular elastic "reclosure element" or "release element" in series with the channel, although the nature of these elements is not known [36-38].

Recent evidence suggests that the tip link is composed of cdh23 and PCDH15 [39-42], which are both members of a membrane adhesion glycoprotein family with cytoplasmic domains containing no significant homology to any other known proteins [43,44]. Although some data indicate that cdh23 is a developmental protein that disappears shortly after the onset of hearing [45], mutations in cdh23 disrupt hair-bundle organization and give rise to deafness and vestibular dysfunction in waltzer mice [43]. *Cdh23 *is also a gene associated with age-related hearing loss [43]. Similar to mice, different mutations in the human *cdh23 *gene can cause DFNB12 and Usher syndrome 1D [46,47]. Hence, the tip link is indispensable for hearing function [48]. Although tip link-associated proteins will be important components of the MET apparatus, hair cells make up a small percentage of the cell population in the cochlea [49], implying that many of these components may be expressed at extremely low levels. Therefore, gene products associated with MET-apparatus components could remain undetected when the entire cochlea or the organ of Corti is used as source material for either RNA or protein investigations. Furthermore, many proteins identified via high-throughput systems (either RNA or protein-based) do not have conserved functional domains indicating their function [50]. These obstacles make searching for MET-components challenging. Lacking knowledge about protein components in the MET apparatus limits our understanding of normal and impaired cochlear physiology.

Several methods have been developed to identify protein-protein interactions. For example, proteomics combines mass spectrometry with co-immunoprecipitation. A major advantage of this approach is the ability to identify physiologically relevant protein-protein interactions that exist within stereocilia *in vivo *[51]. Disadvantages such as low sensitivity and high cost make this approach technically challenging when searching for extremely low-level proteins like MET apparatus components. Alternatively, a genetic approach such as the yeast two-hybrid system, is extremely sensitive and, therefore, suitable for identifying low-abundance protein partners. However, the conventional nucleus-based yeast two-hybrid system requires that protein-protein interactions occur in the nucleus where membrane proteins such as prestin and cdh23 do not reside. In order to overcome these obstacles, we adopted a membrane-based yeast two-hybrid system developed by the Stagljar group [52], in which the transmembrane region and cytoplasmic tail(s) of targeted proteins were used as bait. This system permits identification of proteins that are in the cytoplasm and/or in the cell membrane. Because the bait contains the entire transmembrane region and cytoplasmic tail(s), it will better preserve the native three-dimensional structure of a given protein than does use of the cytoplasmic tail alone as in the conventional nucleus-based yeast two-hybrid system. For this reason, partners identified using the membrane-based approach are more likely to reflect potential *in vivo *interactions. Like other yeast two-hybrid systems, this screen can generate a great number of false positive clones that often bury real signals. Therefore, we built an OHC cDNA library to reduce physiologically irrelevant partners. Using OHC cDNA as source material further increases the sensitivity and decreases false positives. Because cdh23, a component of stereocilia-based cochlear amplification, is located at the apical membrane (tip of hair bundles) [43], and prestin, the agent of somatic electromotility-based cochlear amplification, is at the basolateral membrane [17], we expect that they will have different associated proteins. Identifying and understanding the interactions between each of these two proteins and their potential partners contributes to our knowledge of OHC-based cochlear amplification and mechanoelectrical transduction. It also allows for the possible identification of new deafness-related genes, thereby enabling other investigators to manipulate their functions for therapeutic purposes through molecular biological strategies, pharmacological treatments, and/or gene therapies.

## Results

In order to identify cdh23 and prestin-associated proteins, we used a membrane-based yeast two-hybrid screening process [52] to pull out potential cdh23 and prestin partners from an OHC cDNA library. Because OHCs make up a very small portion of the cell population in the cochlea (~5%) [49], gene products could remain undetected when the entire cochlea or OC is used as source material. For example, a mouse OC library was built from 364 OC samples. Over 20,000 independent clones were isolated from this library (NbLib0053) [53]. Surprisingly, however, prestin was not among the clones in spite of the fact that it is an abundantly expressed OHC-specific gene product. Therefore, in order to eliminate physiologically irrelevant false positive clones and increase sensitivity during library screening, we built a mouse OHC cDNA library suitable for working with a membrane-based yeast two-hybrid system.

### 1. Generation of yeast clones expressing the prestin-bait

**Figure 2:**
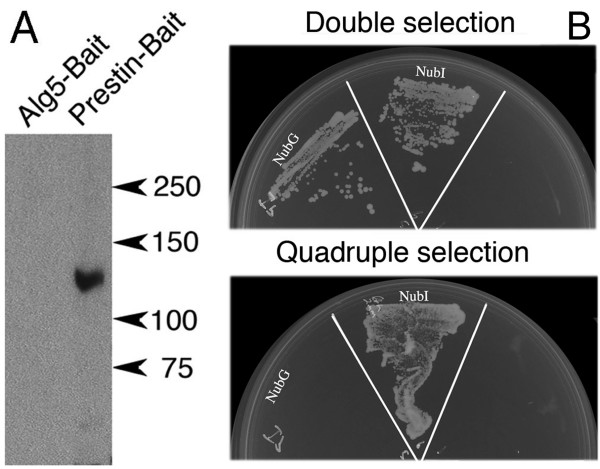
**Analysis of prestin-bait expressing yeast**. (**A**). Expression of the mPrestin-Cub-LexA-VP16 bait fusion protein (~120 KDa) in yeast was verified by SDS-PAGE/Western blot analysis using anti-prestin. (**B**). Both negative and positive control prey proteins were expressed in prestin-bait yeast as demonstrated by their growth on the SD-LT plate. Prestin interacted with the positive control prey (NubI), as indicated by its growth on the SD-LTH plate, but not with the negative control prey (NubG). These data suggest that prestin-protein bait is expressed in the correct orientation with the Cub-LexA-VP16 accessible to the NubG tag of the prey protein and that NubG is not able to reconstitute ubiquitin with mPrestin-Cub-LexA-VP16.

We inserted *Prestin *cDNA into the bait vector pAMBV4 through an *in vivo *recombination method. A CUB-LexA-VP16 tag was attached at the end of prestin. The bait vector carries the LEU2 gene for auxotrophic selection. The Prestin-bait construct, Prestin-LexA-VP16, was transformed into yeast strain NMY51 (Dualsystems Biotech) and prestin-bait expressing yeast clones identified. The sequence of the prestin-bait vector was confirmed through DNA sequencing (data not shown). Western blots in Figure 2A show that prestin protein is only found in the prestin-bait yeast clone. Since there is no homologous prestin gene in yeast, the absence of a prestin band is expected in the control bait yeast (Alg5-Bait).

In order to insure that the prestin bait expressed in yeast can interact with prey proteins, prestin-bait yeast was transformed with the positive prey control plasmid NubI-Alg5 (Dualsystems Biotech). Alg5 is a transmembrane protein with both N and C termini in the cytoplasm, which synthesizes dolichol-phosphoglucose from dolicholphosphate and UDP-glucose. Because Alg5 is not known to interact with the oligosaccharyltransferase complex [54], it should not partner with a glycoprotein like prestin. Without needing the interaction between prestin bait and Alg5 prey, NubI (WT) from the NubI-Alg5 fusion protein can bind Cub from prestin bait as long as the Cub-LexA-VP16 moiety of the prestin bait is also present on the cytosolic side of the membrane. The co-expression of NubI-Alg5 with prestin bait results in reconstitution of split-ubiquitin and activation of the receptor genes. Such actions allow prestin/NubI-Alg5 yeast to grow on the selective quadruple medium (SD-LTHA) as shown in Figure 2B and to show blue color indicating β-Galactosidase degradation by the reporter gene *lacZ *(data not shown here). For a negative prey control, NubG-Alg5 was co-transformed with prestin bait. As expected, prestin/NubG-Alg5 did not grow on the quadruple dropout selective medium (SD-LTHA, Figure 2B), although both prestin and Alg5 were co-expressed by yeast as demonstrated on a double selection plate (SD-LT). Prestin bait alone cannot survive on either the double or quadruple dropout medium. The double dropout (SD-LT) medium is used to select yeast that has both the bait and prey plasmid. The quadruple dropout (SD-LTHA) medium is used to select yeast that has both the plasmids as well as for the interaction of the hybrid proteins encoded by them. These data suggest that Prestin bait was synthesized and inserted into the yeast membrane with the correct orientation, i.e., with its Cub-LexA-VP16-tag facing the cytoplasm. By enabling the use of full-length prestin bait, the system is able to screen for protein-protein interactions at all domains of prestin's 3-dimensional binding structure simultaneously, thereby increasing the sensitivity and specificity of partner protein identification.

### 2. Generation of yeast clones expressing cdh23 bait

**Figure 3:**
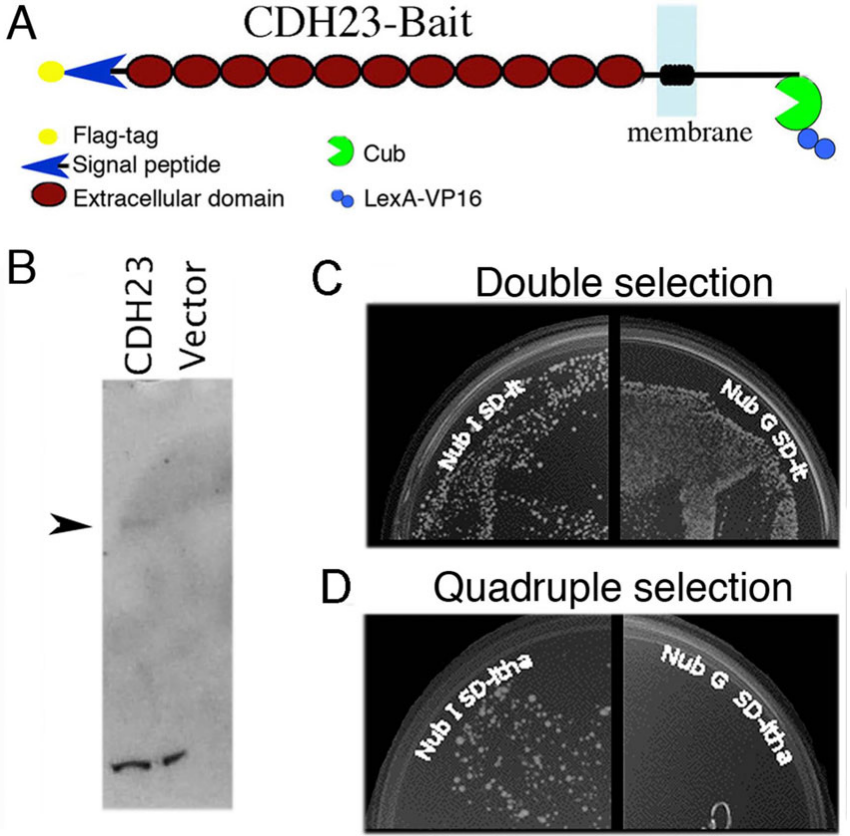
**Analysis of cdh23-bait expressing yeast**. (**A)**. Cartoon of the cdh23-bait construct. (**B**). Western blot of cdh23-bait expressing yeast blotted with anti-FLAG. Cdh23-bait expressing yeast (cdh23) were compared with yeast carrying the empty pTMBV4 vector (vector). The arrowhead indicates the expected cdh23 band. (**C-D)**. The membrane-based yeast two-hybrid analysis for correct expression of the "bait". Cdh23-bait is co-expressed with the positive control prey construct NubI-Alg5 (left side), or the negative control construct NubG-Alg5 (right side) on the double dropout selection medium (SD-LT) (**C**) and quadruple dropout selection medium (SD-LTHA) (**D**).

Similar to prestin bait, we inserted a partial sequence of cdh23 cDNA into the bait vector pTMBV4 through an *in vivo *recombination method. The insert contained the entire cytoplasmic tail, transmembrane domain, fourteen extracellular (EC) domains, a signal peptide and a FLAG-epitope tag at the N-terminus, as shown in Figure 3A. FLAG-tag is used to identify the expression of cdh23 protein, while the signal peptide allows the N-terminus of cdh23 to be located extracellularly. Cub and receptor gene LexA-VP16 were attached at the C-terminus of cdh23, i.e., intracellularly. The cdh23-bait construct was then transformed into yeast strain NMY51 and cdh23-bait expressing yeast clones identified. Although not shown here, the plasmid containing the cdh23-bait construct was also isolated from the yeast for sequencing in order to demonstrate that cdh23 was correctly inserted into the bait vector pTMBV4. Western blots in Figure 3B show that cdh23 protein is only found in the cdh23-expressing yeast clone, not in the control yeast (vector). Like prestin bait, cdh23-bait yeast were transformed with the positive control prey NubI-Alg5 and the negative control NubG-Alg5 prey, respectively. As shown in Figure 3C and 3D, cdh23 bait interacts with NubI-Alg5 prey and grows on quadruple selection media (SD-LTHA) as shown in Figure 3D, but not with the negative control NubG-Alg5 prey, although both cdh23 and Alg5 were co-expressed by yeast as demonstrated in Figure 3C (SD-LT, double selection). These data suggest that cdh23 bait is correctly expressed in yeast with its Cub-LexA-VP16-tag facing the cytoplasm, allowing it to interact with prey proteins. The correctly expressing cdh23-bait construct is the foundation for successful identification of potential cdh23-associated proteins in the membrane-based yeast two hybrid system.

### 3. Screening the OHC library with prestin and cdh23 bait

The yeast two-hybrid system requires little individual optimization and is well suited to screen multiple potential partners in a high-throughput format. In the library screen, auxotrophic selection is achieved through the use of the HIS3 marker. This marker is sensitive but quite leaky, meaning that a bait with a very low level of self-activation may be suitable for screening but could yield high numbers of interacting clones, many of which will turn out to be false positives. Background growth due to leaky *HIS3 *expression was suppressed by adding 3-aminotriazole (3-AT), a competitive inhibitor of the *HIS3 *gene product, to the selection media. Cdh23- and Prestin-bait yeast were co-transformed with empty pDL2-Nx and pDL2-xN vectors, respectively. The survival rates were assayed on quadruple selection plates (SD-LTHA) containing increasing amounts of 3-AT. For cdh23-bait, 2.5 mM 3-AT was required to inhibit self-activation from cdh23-bait/pDL2-Nx vector; for prestin-bait/pDL2-xN yeast, 1 mM 3-AT was required to inhibit self-activation, and for prestin-bait/pDL2-Nx, 2.5 mM 3-AT was required. Although prestin-bait was first transformed using the OHC-pDL2-xN library, the efficiency of transformation was consistently low even after several attempts and few positive clones were identified. The low efficiency and low positive clones were probably due to stop codons at the 3'-ends of the inserts, which break the linkage to Cub-LexA-VP16 tag. Hence, this library was not used for further study.

**Figure 4:**
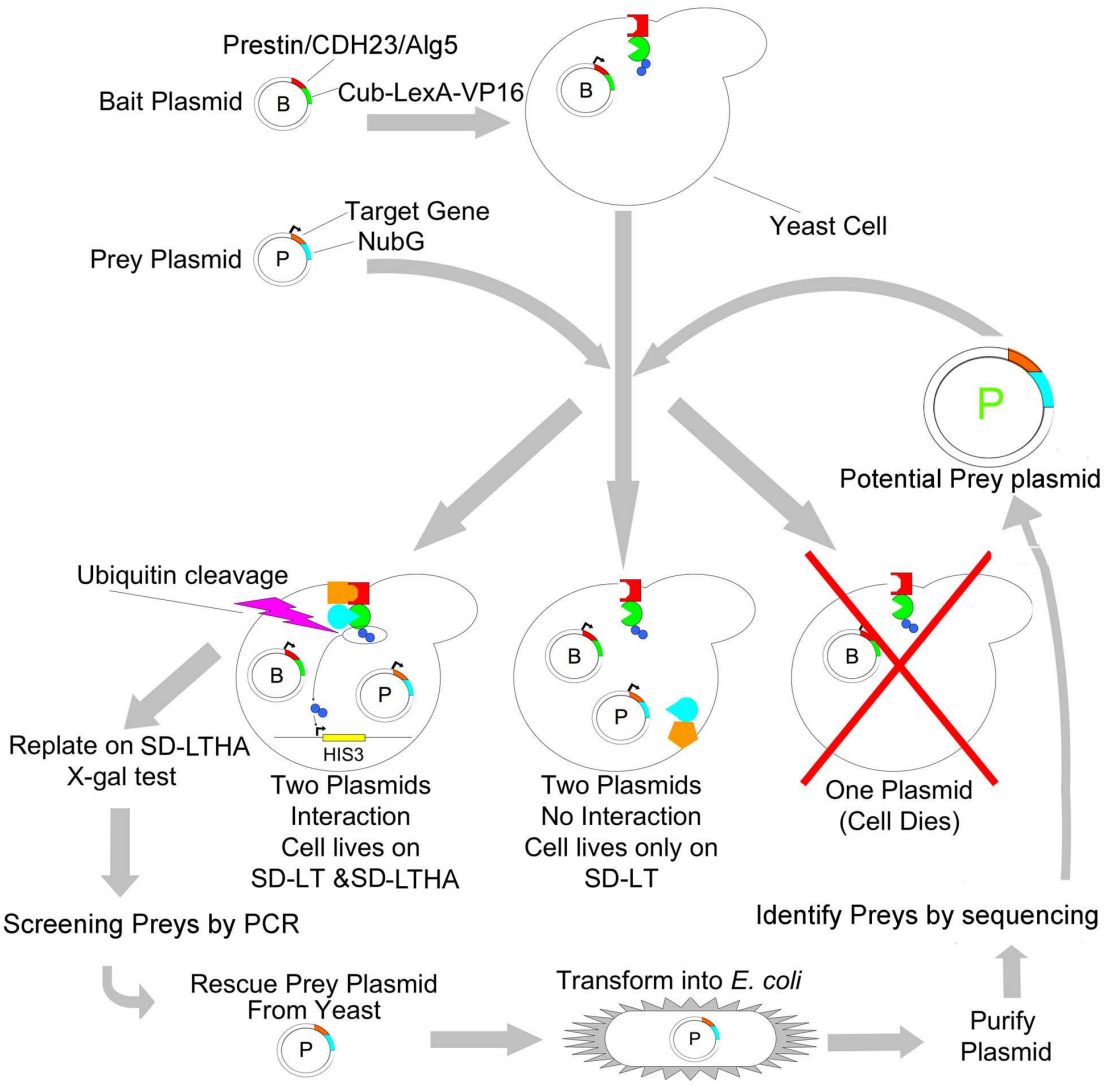
**The flow chart used to screen the OHC library and steps for eliminating false positive clones**. Yeast cells are transformed with bait plasmids containing the main gene of interest: Prestin, cdh23 or Alg5 (control bait) and with prey plasmids containing genes from the OHC library. If only one plasmid is transformed into the cell, the cell will die. If both prey and bait plasmids are transformed, but no interaction takes place between the resulting proteins, which would trigger the reconstitution of ubiquitin, the cell will live on double dropout plates but not on quadruple dropout plates. If prey and bait plasmids are transformed and there is an interaction between the resulting proteins, the cell will live on both double dropout and quadruple dropout plates. The colonies that grew on the quadruple dropout plates were then screened for false positives by replating on quadruple dropout plates containing X-gal, which turns blue in the presence of LacZ. Positive clones were screened by PCR. After prey plasmids were isolated from yeast and transformed into *E. coli*, they were purified and sequenced. Clones of interest were then retransformed into yeast cells along with the bait plasmid in order to confirm their interaction.

The screening process using the OHC-pDL2-Nx library is illustrated in Figure 4. In this case, 7 μg of OHC-pDL2-Nx library DNA was transfected into cdh23- and prestin-bait yeast with a transfection efficiency of ~3.7 × 10^5 ^and 4.8 × 10^5 ^cfu/μg respectively, high enough for each potential partner gene to be independently represented multiple times. Interactors were selected on the quadruple selection (SD-LTHA) plates containing 2.5 mM 3-AT. Several hundred yeast colonies that grew from this initial screen were then re-plated on SD-LTHA/3-AT selection plates. All of them were Lac-Z positive. Approximately 400 clones from cdh23-bait screening and ~300 clones from prestin-bait screening were selected for PCR. Primer pairs were chosen from both ends of the inserts, which allows PCR to amplify the entire OHC cDNA insert. This method eliminates empty or multiple insert clones as it did for the OHC-IHC subtracted library [50]. The PCR screening step significantly reduced false clones and saved a great deal of unnecessary labor. Yeast with only one insert cDNA band (size larger than ~500 bp) were then cultured on SD-LT selection media. Their plasmids were isolated and transformed into *E. coli *strain XL-1 blue. The plasmid isolated from the yeast was a mixture of the bait plasmid (cdh23 or prestin) and one type of OHC cDNA insert plasmid. Since the bait plasmid does not have ampicillin-resistant selection but the prey cDNA construct does, the transformant containing the OHC cDNA insert was selected on an ampicillin-containing LB plate (LBA). The plasmid was then isolated and its identity determined by DNA sequencing.

Like other genetic selection methods, the membrane-based yeast two-hybrid assays isolated a certain number of false positives showing *His*^+ ^and *lacZ*^+ ^phenotypes, independent of any interaction with cdh23 or prestin. These false positive clones include the proteins normally found only in nuclei, such as transcription factors, and were therefore eliminated. False positive clones were also eliminated by transforming the isolated prey plasmid (isolated from *E. coli*) with the positive bait (prestin or cdh23) and the control bait Alg5, respectively. True partner proteins yield *His*^+ ^and *lacZ*^+ ^phenotypes when co-expressed with either bait (cdh23 or prestin) but not with the control. After the above steps were taken to weed out false positives, 45 clones associated with 18 independent genes, were identified as potential cdh23 partners. 48 clones associated with 28 independent genes, were identified to be potentially associated with prestin. The two groups of potential partners are completely different from each other, sharing none of the same proteins.

**Figure 5:**
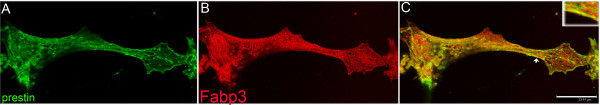
**Co-localization of prestin and Fabp3 in OK cells**. OK cells were transiently co-transfected with GFP-prestin and Xpres-tagged Fabp3. After 48 hrs, cells were fixed and incubated with mouse anti-Xpress followed by the corresponding secondary antibody. Yellow image (C) is superimposed from green prestin (A) and red Fabp3 (B) images, indicating the co-localization of prestin and Fabp3. For better visualization of the co-localization, the demarcated portion (indicated by arrowhead) of panel C is shown in the left corner of panel. Bar: 23.8 μm.

Because yeast and mammalian cells differ in many ways, the detection of an interaction between prestin/cdh23 and their potential partners in yeast does not necessarily mean that the same interaction will occur in mammalian cells [55]. Therefore, in order to evaluate the interactions between prestin/cdh23 and potentially associated proteins, the coding sequences of some of the potential partners were inserted into mammalian expressing vector pcDNA 3.1/HisC. Plasmids encoding these potential partners were transiently co-transfected with prestin or cdh23 into an opossum kidney (OK) mammalian cells line. Figure 5 shows an example of the co-localization expression pattern between bait and prey. Fatty acid binding protein 3 (Fabp3) is a potential prestin-partner. When Fabp3 and GFP-prestin were co-expressed in OK cells, Fabp3 staining (red) co-localizes with GFP-prestin (Figure 5). These data are consistent with the fact that Fabp3 does interact with prestin in yeast. In other words, potential prestin/cdh23 partners identified from yeast are capable of interacting with their bait in mammalian cells. It should be noted, however, that co-localization experiments are the first in a sequence of steps required to verify the interaction between prey and bait in a mammalian cell system. In order to understand the physiological significance of the interaction, additional investigations involving both *in vitro *biochemical experiments and *in vivo *physiological investigations are required for each potential partner.

Among potential cdh23 partners, the most abundant group (25 of the 45 clones, 55%) has an EF-hand motif, which is a calcium-binding domain. These proteins belong to five different genes, which code for: calmodulin (CaM), oncomodulin, parvalbumin, EHD4, and S100 calcium binding protein A1 (S100A1). S100A1, however, is only expressed in supporting cells [56], which are also present in the OHC-rich library due to their unavoidable inclusion during the OHC collection process [57]. Oncomodulin is a small calcium-binding protein related to β-parvalbumin that was originally found in malignant neoplasms and placenta, and has been classified as an oncodevelopmental protein [58]. However, OHCs are the only postnatal, adult, non-malignant tissue that expresses oncomodulin [59]. Previous reports also indicate that CaM, parvalbumin and EHD4 are all expressed in hair cells [60-63]. The observation that the majority of cdh23's potential partners contain a calcium-binding domain is interesting since the intracellular domain of cdh23 is located where calcium concentration is highly regulated. In fact, Ca^++ ^is a crucial element for fast/slow adaptation and cilia-based amplification even though there is no universal agreement about the mechanisms of its action [25,27,33,64]. Discovery of an interaction between CaM and cdh23 may be a novel and crucial step for understanding the molecular basis for adaptation. For example, cdh23 could be the intracellular elastic "reclosure element" or "release element" predicted by several models to be in series with the MET channel [36-38].

Among potential prestin binding proteins, the most abundant group (18 of 48 clones, 38%) comprised electron transport proteins including cytochrome *b*, subunits of NADH-ubiquinone oxidoreductase, and ATP synthase 6. At first glance, these potential prestin-associated proteins appear to be physiologically irrelevant false positive clones. However, OHCs that lack prestin, as well as OHCs that lack fully *functional *prestin, show significant cell death compared to their wildtype littermates [18,23]. Plasma membrane electron transport systems have been implicated in various functions including the prevention of cell death (for a review see [65]). Hence, the close association between prestin and proteins involved in electron-transport systems leads us to suspect that these electron transport proteins may play an important role in OHC survival and could be dependent on prestin's function. Since a large portion of cDNA from OHCs was derived from mitochondrial genes [66] (55% of known gene clones), we tested whether these mitochondrial clones were false positives, showing *His*^+ ^and *lacZ*^+ ^phenotypes, independent of any interaction with prestin. First, we used cdh23 as the "bait" to screen the OHC library. A group of prey proteins, which differ completely from prestin-associated potential partners, were identified. As noted above, the most abundant clones (55%) were proteins containing calcium-binding domains, which were never found in the prestin-associated pool. Most importantly, not one of the cdh23-partner proteins is associated with electron transport. Second, individual plasmids of potential prestin partners were isolated and transformed into the prestin-bait and the cdh23-bait yeast individually. True prestin partners yield *His*^+ ^and *lacZ*^+ ^phenotypes when co-expressed with the prestin-bait, but do not express these phenotypes with the cdh23-bait. None of the electron transport proteins interacted with cdh23-bait but all interacted with prestin-bait. These two data sets suggest that prey identified through the membrane-based yeast two-hybrid is indeed bait-dependent. In other words, mitochondrial proteins do interact with prestin in the membrane-based yeast two-hybrid system.

Anatomical results indicate that mitochondria are clustered along the lateral wall of the OHC, as if prestin or something related to its function, has a special metabolic need [67]. This arrangement contrasts with that in inner hair cells where the mitochondria are scattered throughout the cytoplasm. Although mitochondrial proteins do not usually interact with PM proteins like prestin due to their spatial separation, there are two possible explanations for the association demonstrated here. First, mitochondrial proteins are prestin partners because the OHC's unique cellular structure may allow mitochondrial proteins to be inserted into cellular membranes. For example, Hensen's body, which contains rich mitochondria, was found only in OHCs [68]. Although the function of Hensen's body is not known, it has been suggested that this structure is involved in protein recycling [69]. It is, therefore, conceivable that one of the functions of the unique Hensen's body is to deliver mitochondrial proteins into the OHC's PM. Second, it is possible that these mitochondrial proteins are physiologically irrelevant. However, the fact that prestin directly interacts with proteins involved in the electron transport system implies that prestin may interact with proteins similar in structure to mitochondrial proteins. We have also identified several unknown proteins. These proteins could potentially act as electron transport proteins located at the PM. Therefore, the functions of these unknown proteins require further investigation.

### 5. Known gene products identified as potential partners of cdh23 and prestin

Besides the most abundant group, other proteins with known functions are listed in Table 1. Eight potential partners of cdh23 were identified. Among them, otospiralin (Otos) [70] and gap junction protein, beta 6 (Gjb6) [71] are not expressed in hair cells, making them physiologically irrelevant. Endosulfine alpha (ENSA) is a member of the cyclic adenosine monophosphate (cAMP)-regulated family of phosphoproteins [72], which modulates ATP-dependent potassium (KATP) channels [73]. Protein tyrosine phosphatase, receptor type, A (Ptpra) is a Src family kinase activator and mediator of multiple biological effects [74], while symplekin is a transcription factor known to be associated with junctional components in order to regulate gene expression [75]. Lastly, twinfilin is a highly conserved actin-binding protein that regulates cytoskeletal dynamics in organisms from yeast to mammals [76]. Whether these proteins are indeed cdh23-binding proteins needs further investigation. Nevertheless, this group of proteins is largely associated with modification, either at the protein or gene regulation level.

**Table 1:** Potential prey proteins with known functions

**Prestin prey**	**Cdh23 prey**
Tetraspanin 6 (Tspan 6) (**BC003733.1**)	Protein tyrosine phosphatase, receptor type, A (Ptpra) (**NM_008980.1**)

CD9 antigen (CD9, Tspan29) (**BC070474.1**)	Endosulfine alpha (ensa) (**AK006149.1**)

CD52 antigen (**AK155728.1**)	Symplekin (**BC049852.1**)

Emopamil binding protein-like (Ebpl) or emopamil binding related protein (Ebrp) (**BC027422.1**)	Heat shock protein 5 (Hspa5) (**NM_022310.2**)

Potassium intermediate/small conductance calcium-activated channel, subfamily N, member 2 (Kcnn2) (**AK050390.1**)	Heat shock protein 8 (Hspa8) (**NM_031165.4**)

Solute carrier family 35, member B1 (SLC35B1) (**NM_016752.1**)	Twinfilin, actin binding protein, homolog 1 (**BC015081.1**)

Fatty acid binding protein 3, muscle and heart (Fabp3) (**AK142156.1**)	Gap junction protein, beta 6 (Gjb6) (**NM_008128.3**)

β-2 microglobulin (B2M) (**BC085164.1**)	Otospiralin (Otos) (**NM_153114.2**)

Bone gamma carboxyglutamate protein 1 (Bglap1) (**NM_007541.2**)	

Frizzled-3 (FZ3) (**NM_021458**)	

Vapa (Vesicle-associated membrane protein associated protein A) (**NM_013933**)	

Dynein light chain Tctex-type 1 (Dynlt1) (**NM_009342.2**)	

For potential prestin-associated proteins with known functions, some were associated with protein transport, such as vesicle-associated membrane protein, associated protein A (VAPA). VAPA is an integral membrane protein localized in either intracellular vesicles or at tight junctions in many cells and tissues. It is also reported to be associated with the endoplasmic reticulum and microtubules [77,78]. Frizzled-3 (FZ3), which is localized asymmetrically at the lateral faces of hair cells, may also be involved in the planar orientation of stereociliary bundles in hair cells [79,80]. In fact, most of the potential prestin-associated proteins are membrane proteins including some of the super tetraspanin family such as tetraspanin 6 (Tspan 6) [81] and CD9 antigen (CD9 or Tspan29). A typical tetraspanin has four transmembrane domains. They are distributed in virtually all cell types and involved in various cell-cell and matrix-cell interactions ranging from differentiation to signal transduction [82,83]. Because they can bind groups of protein partners and facilitate their functions, they have been referred to as "molecular facilitators", "molecular organizers", "tetraspanin networks", and "membrane microdomains" [84,85]. Compared to cdh23, prestin partners have a more hydrophobic composition, making them more likely to be membrane proteins.

### 6. Unknown gene products identified as potential partners of cdh23 and prestin

There are a total of 12 gene products with unknown functions identified from prestin- and cdh23-bait screening as listed in Table 2. Some already have names given through bioinformatics such as Tmem59 (Transmembrane protein 59) or ceacam16 (carcinoembryonic antigen-related cell adhesion molecule 16), although no functional information is reported. Other clones are given ID numbers such as RIKEN 1990002N15, RIKEN 5730496F02 and RIKEN 2310057J16. These are unclassified genes with no domains indicating potential function. Table 2 also lists mouse and human chromosomal locations, which match possible associated deafness loci. For example, *ceacam16 *is located at 19q13.31 near the DFNA4 locus. Although mutation in MYH14 can cause DFNA4, there are reports suggesting that another unidentified gene is also involved in this type of deafness [86]. These data suggest that ceacam16 may have an important role in hearing. The RIKEN 2310057J16 gene is located at 19p13.3-13.2 where the loci of DFNB15 [87], DFNB68 [88], DFNB72 [89] and DFNA57 [90] are also found, implying that this gene could be a potential deafness-associated gene.

**Table 2:** Potential prey proteins with unknown functions

**Prestin prey**	**Mouse-chromosome**	**Human-chromosome**	**Deafness-associated Loci**
Yip1 domain family, member 6 (Yipf6) (**NM_207633.2**)	X C2	Xq12	

Tmem59 (**BC058273**)	4 C7 (51.8 cM)	1p36-p31	DFNA2:1p34

Tmem11 (**NM_173453**)	11 B2	17p11.2	

Tmem85 (**NM_026519**)	2 E4	15q14	

Unclassifiable (**AC123616**)	8		

unknown(**AC123616**)	12 C3	14q23.2	

unknown (RIKEN4632425P12 (**AC166991**)	12 C3	14q23.2	

			

**Cdh23 prey**			

Stefin A-like protein (**BC100530.1**)	16B3		

CEACAM16 (carcinoembryonic antigen-related cell adhesion molecule 16) (**NM_001033419.2**)	7A	19q13.31	DFNA4:19q13

RIKEN 5730496F02 (**AK077642**)	18 E3	18q21.1	

RIKEN 2310057J16 (**NM_027171**)	8 A1.1	19p13.3-p13.2	DFNB15:19p13.3-13.1
			DFNB68: 19p13.2
			DFNA57:19p13.2
			DFNB72:19p13.3

RIKEN 11990002N15 (**NM_001033145**)	9 E3.3	3q24	DFNB15:3q21-q25
			OTSC5: 3q22-q24

## Discussion

Knowing the function of proteins and protein interactome networks in hair cells is a crucial first step for understanding hearing. Therefore, identification of important proteins is carried out by diverse strategies. Some use DNA-microarray technology to identify all mRNA from hair cells [91], others aim to identify all proteins located in hair bundles [51]. These high-throughput approaches have generated an enormous amount of data and have the potential to contribute to our understanding of the MET apparatus and OHC function. However, these approaches are not ideal for finding low-level mRNA/proteins such as those that constitute the MET apparatus. In addition, these methods are at a disadvantage when trying to understand the potential functional roles of uncharacterized *de novo *proteins since their associated proteins are unknown. Hence, we strongly favor an approach in which a search is made for partner proteins, especially when identifying MET apparatus components, inasmuch as there is no general agreement on what family of proteins might constitute the MET channel and/or its essential companions [92]. Because prestin and cdh23 perform their physiological functions only in membrane environments, their associated proteins are likely to be membrane bound or cytoplasmic proteins that can associate with prestin or cdh23 located in the plasma membrane. Hence, we chose the membrane-based yeast two-hybrid system to search for their associated proteins. Because this assay is a new innovation [52], there are potential questions regarding its performance. For example, is the membrane-based yeast two-hybrid assay better than the traditional nucleus-based system? In order to answer this question, we compared prey identified using two methods. When the nucleus-based system was used to search for prestin partners, a transcriptional repressor, promyleocytic leukemia zinc finger protein (PLZF), was the only potential prestin-associated protein found when the C-terminus (including stas domain) was used as bait [93]. The physiological significance of this interaction, as stated in the Nagy et al. paper, remains to be explored. In contrast, when full-length prestin was used as bait in the membrane-based system as demonstrated in this paper, numerous unknown/known potential partners were identified. These prey probably bind to prestin in its native three-dimensional structure, including both membrane and non-membrane regions. For cdh23, two proteins, harmonin and MAGI-1, have been identified as cdh23 partners when using the C-terminus as bait [94,95]. Both of these partners are multi-PDZ domain containing scaffold proteins that interact with the non-membrane bound PDZ binding interface (PBI) of cdh23. However, as demonstrated in the current study, a group of completely different cdh23 associated proteins, the calcium-binding proteins, were also found as potential cdh23-partners. We suspect that binding between cdh23 and calcium-binding proteins involves the cdh23's transmembrane region, independent of PDZ/PDI binding sites. Although we do not know the exact binding sites, or how the membrane region affects protein-protein interactions, the information provided in this report advances our understanding of how prestin and cdh23 impact hearing.

Prestin is only found in OHC basolateral membranes while cdh23 is exclusively located in apical membranes, i.e., the stereocilia. Logically and functionally, they should have different associated proteins. When cdh23 and prestin were used as bait to screen the same OHC-rich cDNA library, we have indeed identified two completely different prey groups associated with prestin and cdh23 respectively. Cdh23 is associated with calcium-binding proteins, while prestin is connected with electron-transport proteins. These unanticipated results were never reported when a traditional yeast two-hybrid system was used. Hence, these data demonstrate that the membrane-based yeast two-hybrid system is not just more advanced on a theoretical basis, but it provides more inclusive information regarding interactome components in hair cells.

Compared to other high-throughput systems, our strategy has several advantages. First, it is an extremely sensitive genetic approach, suitable for identifying low-abundance protein partners, which many of the MET-apparatus components are likely to be. Second, because the selection strategy is membrane-based, this system permits identification of proteins that are in the cytoplasm and/or in the cell membrane, while the conventional nucleus-based yeast two-hybrid system requires that protein-protein interactions occur in the nucleus, where membrane proteins such as the MET channels would not be selected. Third, this method would be particularly useful for identifying putative functional roles for uncharacterized proteins by placing them within the functional context of the cdh23 and prestin. Fourth, a bait containing the entire transmembrane region and cytoplasmic tail will better preserve the native three-dimensional structure of a given protein than does the use of the cytoplasmic tail alone as in the conventional nucleus-based yeast two-hybrid system. Furthermore, we built an OHC library to reduce physiologically irrelevant partners. This OHC library was created from cDNA isolated from P11 to P23 mice and, therefore, includes both transitional developing mRNA and mRNA associated with mature OHC functions. Nevertheless, the library excludes a significant amount of physiologically irrelevant gene products. Using OHC cDNA as source material further increases the sensitivity and decreases false positives. By combining an enriched OHC mRNA source and a powerful genetic tool to ferret out protein-protein interactions, we identified several interesting *de novo *gene products that have potentially important roles in hearing. These data confirm that the membrane-based yeast two-hybrid system is a powerful tool for investigating protein-protein interactions.

The membrane-based yeast two-hybrid method identified two different groups of proteins using prestin and cdh23 as bait. Both groups contain potentially significant partners. It is well known that adaptation in hair cells, and potentially amplification as well, is due to MET processes mediated by Ca^++ ^(for review, see [27,32,33]). Because cdh23 is an essential component of the tip link that delivers force to the MET channels [40], our discovery of calcium-binding proteins as cdh23 partners, places them at a critical location where they could mediate transducer function. For example, CaM was found at both ends of the tip link [60]. CaM is known to play important roles in MET and to interact with several components of the channel complex including myosin1c, the motor for slow adaptation [64,96-98]. However, it has never been demonstrated that CaM is associated with cdh23. Further investigation of these unexpected and suggestive results should assist our understanding of the molecular basis of transduction and possibly of fast adaptation. In contrast, the discovery of abundant electron transport proteins related to the molecular motor prestin, raises the hope for an explanation of the observations gained from knockout/knockin animals that the presence of functional prestin is required for OHC survival. These two discoveries, using this new methodology, open potentially fruitful lines of investigation into MET function and OHC death.

## Conclusion

Two prey groups, very different from each other, have been identified by using prestin and cdh23 as bait. Cdh23 prey are dominated by calcium-binding proteins. This unanticipated result makes sense considering the function and the environment of cdh23. Most of prestin-associated proteins are involved in electron transport proteins. This unforeseen result implies a potential function of prestin in addition to its role in cochlear amplification. Furthermore, a group of *de novo *genes closely associated with deafness loci were also identified.

## Methods

### Antibodies

The rabbit anti-C-terminus mPres (anti-C-mPres) polyclonal antibody [99] was used in a 1:2000 dilution for immunofluorescence and Western blots. An anti-FLAG (Sigma) antibody was used in a 1:1000 dilution in Western blot. Anti-Xpress antibody from Invitrogen (Carlsbad, CA) was used at 1:200 in the immunofluorescence experiments. Secondary antibodies used include goat anti-mouse IgG-Alexa Fluor 546 (Molecular Probes, Eugene, OR); Donkey anti-rabbit IgG-HRP (horseradish peroxidase) and donkey anti-mouse IgG-HRP were purchased from Pierce or Jackson ImmunoResearch.

### Building the OHC-cDNA library

All surgical and experimental procedures were conducted in accordance with the policies of Northwestern University's Animal Care and Use Committee. After the animal was killed with an overdose of anesthetic (Euthasol 200 mg/kg), cochleae from mice ranging in age from P11–P23 were dissected in L-15 medium (Sigma). About 10,000 OHCs were collected for building the library. The distribution of these OHCs among different ages of OHCs were: P11 (3.5%), P12 (4%), P13 (4%), P14 (3.5%), P15 (5%), P17 (25%), P18 (8%), P19 (22%) and P23 (25%). The detailed procedure for OHC isolation and cDNA creation was published recently [57]. Briefly, an OHC cDNA pool was created by reverse transcription using PowerScript™ reverse transcriptase, followed by 22 cycles of amplification with 5'Cap and oligo-dT-dependent SMART PCR primers provided by the Creator™ SMART cDNA Library Construction Kit (Clontech). The OHC cDNA pool was then digested with an *sfi *I restriction enzyme and separated through a CHROMA SPIN-400 column. cDNA fragments with sizes larger than ~200 bp were ligated into pDL2-xN and pDL2-Nx vectors, respectively (Dualsystems Biotech, Switzerland) and transformed into XL10-Gold Ultracompetent cells (Stratagene, La Jolla, CA). cDNA was under the control of an ADH promoter with an ampicillin resistant gene and TRP1 for auxotrophic selection in yeast. The pDL2-Nx vector adds NubG at the N-terminus of every insert cDNA. pDL2-Nx adds NubG at the C-terminus of every insert cDNA. The libraries (OHC-pDL2-xN and OHC-pDL2-Nx) were amplified once. Plasmid DNA containing different cDNAs was isolated from XL10-Gold using Plasmid Midi Kit (Qiagen). The original titers (before library amplification) for OHC-pDL2-Nx and OHC-pDL2-xN libraries were 6.4 × 10^4 ^and 1.7 × 10^5 ^cfu/ml respectively.

### Building cdh23- and prestin-bait

#### Establishing prestin- and cdh23-bait expressing yeast

cDNA encoding mPrestin and cdh23 was amplified using PCR primers that permitted the cloning of mPrestin and cdh23 into pAMBV4 and pTMBV4 bait vectors (Dualsystems Biotech, Switzerland), respectively by *in vivo *recombination directly in yeast. Forward and backward primer sequences were as follows: mPrestin-forward: 5'-AGC TAT ACC AAG CAT ACA ATC AAC TCC AAG CTG GCC GCT CTA GAC AAA AAT GGA TCA TGC TGA AGA AAA TG; mPrestin-backward: 5'-TAA GCT TGA TAT CGA ATT CCT GCA GAT ATA CCC ATG GAG GCC TTT TGC CTC GGG GGT GGT GG; Cdh23-forward: 5'-CTC ATT AGA AAG AAA GCA TAG CAA TCT AAT CTA AGT TTT CTA GAC AAA AAT GTC TGC ACT TCT GAT CCT AG; Cdh23-backward: 5'-TAA GCT TGA TAT CGA ATT CCT GCA GAT ATA CCC ATG GAG GCC TTT CAG CTC CGT GAT TTC CAG AGG. These primers include a 45 bp homology region at the 5' and 3' ends. These 45 bp flaps recombine with identical sequences upstream of the two *Sfi *I sites in pAMBV4 (for prestin) and pTMBV4 (for cdh23) vectors. mPresitn-pcDNA3.1/CT-GFP-TOPO [17] was used as a template for creating the prestin-bait construct. Otocdh23 DF-pFLAG-CMV-1 (kindly provided by Dr. James Bartles) was used as a template for creating the cdh23-bait construct. Otocdh23 DF contains a FLAG-tag, the extracellular cadherin repeats (domains 14–27), the transmembrane domain, and the cytoplasmic tail including the peptide encoded by exon 68. The Advantage 2 Polymerase PCR Kit (BD Bioscience) was used to perform the PCR reaction: 95°C for 1 min, 5 cycles of 95°C for 15 sec, 55°C for 30 sec, 68°C for 3 min, followed by another 25 cycles of 95°C for 15 sec, 68°C for 3 min. The PCR product was run on a 0.8% agarose gel. 2 kb (the full-length prestin cDNA) and 6 kb bands (cdh23 cDNA) were purified using a gel purification kit (Qiagen). The purified mPrestin and cdh23 cDNA and linearized pAMBV4 (cut with *Sfi *I) were co-transformed into yeast strain NMY51 (*MATa his3Δ200 trp1-901 leu2-3, 112 ade2 LYS2::(lexAop)_4_-HIS3 ura3::(lexAop)_8_-lacZ ade2::(lexAop)_8_-ADE2 GAL4*) (Dualsystems Biotech, Switzerland), respectively. Cotransformation results in homologous recombination and gap repair, yielding prestin- and cdh23-bait constructs, which allow yeast growth on SD-Leu selective plates. The prestin- and cdh23-bait plasmids were then isolated from prestin-expressing and cdh23-expressing yeast for sequence analysis. The expression of the mPrestin-Cub-LexA-VP16 fusion protein and cdh23-LexA-VP16 fusion protein were analyzed by LDS-PAGE/Western blot with anti-C-mPres and anti-FLAG, respectively.

#### Testing for the correct expression of prestin and cdh23 proteins in yeast

Prestin- and cdh23- expressing yeast were cultured in SD-Leu media at 30°C over night until they reached an OD_546 _of 0.6. 2 μg each of pAlg5-NubI and pAlg5-NubG plasmids were transformed into prestin- and cdh23-expressing yeast according to the manufacturer's instructions (DUALmembrane kit. Biotech, Switzerland). Half of the transformed yeast were cultured on the double dropout (SD-leu-trp, i.e., SD-LT) medium, while the other half were cultured on the quadruple dropout (SD-leu-trp-his-ala, i.e., SD-LTHA) medium. Yeast-growth data were collected after incubation at 30°C for 2–3 days.

#### 3-AT titration

DNA of pDL2-xN and pDL2-Nx vectors (no inserts) was transformed into prestin- and cdh23-expressing yeast, respectively. The co-transformed yeast were cultured on SD-LTHA plates containing different 3-AT concentrations. Data regarding yeast growth were recorded 2–4 days post transformation.

### Library screening for interactors

All necessary controls and references (to the Stagljar group's pioneering work) are described in the manufacturer's manual (DUALmembrane kit, Biotech, Switzerland). 7 μg of OHC-pDL2-Nx library DNA was transformed into cdh23- and prestin-bait yeast, respectively. The co-transformed yeast were also plated on SD-LT plates for calculating the transformation efficiency. After 3 days incubation at 30°C, hundreds of interactor clones were collected from SD-LTHA plates and restreaked on SD-LTHA +2.5 mM 3-AT plates. After incubating at 30°C for 2–3 days, the X-Gal staining assay was performed according to the company's manual. His^+ ^and lacZ^+^positive clones were used to perform PCR. Small amounts of yeast from the plates were mixed with a PCR reaction solution containing forward primer: 5'-ggaatccctggtggtccatac and backward primer: 5'-gcg tcc caa aac ctt ctc aag c. This pair of primers allows PCR to amplify entire OHC cDNA inserts. Taq (Sigma) was used to perform the PCR reaction: 94°C for 3 min, 30 cycles of 94°C 30 sec, 56°C 30 sec, 72°C 1 min. The PCR product was run on 1% agarose gel. Yeast with only one insert cDNA-band (size larger than ~500 bp) were then cultured on SD-LT selection media. Their plasmids were isolated and transformed into *E. coli *strain XL-1 blue (Stratagene) and grown on LBA plates. The plasmids were isolated from XL-1 blue and their identity determined by DNA sequencing. The isolated plasmids (prey) with unique gene products were co-transformed back into the positive bait (prestin or cdh23) and the control bait pMBV-Alg5 (Alg5-bait), respectively.

### LDS-PAGE/Western blot

For prestin and cdh23 expression analysis, pellets of prestin- and cdh23-bait yeast were mixed with 2× LDS (lithium dodecyl sulphate) Laemmli sample buffer, plus 100 mM DTT, protease inhibitor cocktail (1:50, Sigma P8340), 100 μg/ml PMSF (Sigma) and DNase (10 μg/ml). Acid-washed glass beads (420–500 μm) were added to break cell walls. After separating nuclei, unlysed cells and glass bead, samples were loaded and run on a 4–20% Precise gel (Pierce). LDS was used instead of SDS because the latter precipitates in the cold [100]. After separation, the gel proteins were electrotransferred onto a nitrocellulose or Immobilon-P transfer membrane (Millipore), blocked with 2% non-fat dry milk and 2% BSA. Anti-C-mPres was used to detect prestin-expressing bait; anti-FLAG to detect cdh23-expressing bait. Donkey anti-rabbit IgG-HRP or anti-mouse IgG-HRP were the corresponding secondary antibodies. Immunoreactive bands were visualized with the ECL Western blotting detection system (Pharmacia).

### Cell culture and immunofluorescence experiments

Prey cDNA were cut from pDL2-Nx vectors by BamHI/EcoRI and inserted into pcDNA3.1/HisC, which has a Xpress-tag at the N-terminus of prey cDNA. Constructs encoding GFP-tagged prestin have been described previously [101]. Plasmids encoding Xpress-prey were transiently co-transfected with GFP-prestin in opossum kidney (OK) cells according to the protocols described in Zheng *et al*. [101]. The transiently transfected cells were fixed with 1% formaldehyde in PBS for 10 minutes at room temperature 44–48 hours after transfection. After blocking in PBS with 5% BSA and 0.1% saponin for 1 hour at room temperature, the cells were incubated with monoclonal anti-Xpress in PBS with 5% BSA and 0.1% saponin for 2 hours at room temperature, following by secondary antibody, goat anti-mouse IgG-Alexa Fluor 546 (1:400). The samples were mounted on glass slides with Fluoromount-G (Southern Biotechnology Associates, Inc., Birmingham, AL) and observed using a Leica confocal system with a standard configuration DMRXE7 microscope.

## Abbreviations

OHCs: Outer hair cells; IHCs: inner hair cells; cdh23: Cadherin 23; OC: organ of Corti; MET: mechanoelectrical transduction; KO: knockout; KI: knock-in; PM: plasma membrane; PCDH15: protocadherin 15; UBPs: ubiquitin-specific proteases; CaM: calmodulin; S100A1: S100 calcium binding protein A1; VAPA: vesicle-associated membrane protein, associated protein A; ceacam16: carcinoembryonic antigen-related cell adhesion molecule 16; LDS: lithium dodecyl sulphate.

## Authors' contributions

JZ and CTA created OHC-cDNA libraries. CTA also screened the library with prestin bait. KKM screened the library with cdh23-bait. MAC and PD conceived the project and contributed to the writing of the manuscript. JZ collected the data and directed the project. All authors read and approved the final manuscript.
